# Interactions between *Asaia*, *Plasmodium* and *Anopheles*: new insights into mosquito symbiosis and implications in Malaria Symbiotic Control

**DOI:** 10.1186/1756-3305-6-182

**Published:** 2013-06-18

**Authors:** Aida Capone, Irene Ricci, Claudia Damiani, Michela Mosca, Paolo Rossi, Patrizia Scuppa, Elena Crotti, Sara Epis, Mauro Angeletti, Matteo Valzano, Luciano Sacchi, Claudio Bandi, Daniele Daffonchio, Mauro Mandrioli, Guido Favia

**Affiliations:** 1 Scuola di Bioscienze e Biotecnologie, Università degli Studi di Camerino, Camerino 62032, Italy; 2 Department of Food, Environmental and Nutritional Sciences, Università degli Studi di Milano, Milan 20133, Italy; 3 Dipartimento di Patologia Animale, Igiene e Sanità Pubblica Veterinaria, Università degli Studi di Milano, Milan 20133, Italy; 4 Dipartimento di Biologia Animale, Università degli Studi di Pavia, Pavia 27100, Italy; 5 Dipartimento di Scienze della Vita, Università degli Studi di Modena e Reggio Emilia, Modena 41125, Italy

**Keywords:** *Asaia*, *Plasmodium*, *Anopheles*, Malaria, Haemocytes

## Abstract

**Background:**

Malaria represents one of the most devastating infectious diseases. The lack of an effective vaccine and the emergence of drug resistance make necessary the development of new effective control methods. The recent identification of bacteria of the genus *Asaia*, associated with larvae and adults of malaria vectors, designates them as suitable candidates for malaria paratransgenic control.

To better characterize the interactions between *Asaia, Plasmodium* and the mosquito immune system we performed an integrated experimental approach.

**Methods:**

Quantitative PCR analysis of the amount of native *Asaia* was performed on individual *Anopheles stephensi* specimens. Mosquito infection was carried out with the strain PbGFP_CON_ and the number of parasites in the midgut was counted by fluorescent microscopy.

The colonisation of infected mosquitoes was achieved using GFP or DsRed tagged-*Asaia* strains.

Reverse transcriptase-PCR analysis, growth and phagocytosis tests were performed using *An. stephensi* and *Drosophila melanogaster* haemocyte cultures and DsRed tagged-*Asaia* and *Escherichia coli* strains.

**Results:**

Using quantitative PCR we have quantified the relative amount of *Asaia* in infected and uninfected mosquitoes, showing that the parasite does not interfere with bacterial blooming. The correlation curves have confirmed the active replication of *Asaia*, while at the same time, the intense decrease of the parasite.

The ‘in vitro’ immunological studies have shown that *Asaia* induces the expression of antimicrobial peptides, however, the growth curves in conditioned medium as well as a phagocytosis test, indicated that the bacterium is not an immune-target.

Using fluorescent strains of *Asaia* and *Plasmodium* we defined their co-localisation in the mosquito midgut and salivary glands.

**Conclusions:**

We have provided important information about the relationship of *Asaia* with both *Plasmodium* and *Anophele*s. First, physiological changes in the midgut following an infected or uninfected blood meal do not negatively affect the residing *Asaia* population that seems to benefit from this condition. Second, *Asaia* can act as an immune-modulator activating antimicrobial peptide expression and seems to be adapted to the host immune response. Last, the co-localization of *Asaia* and *Plasmodium* highlights the possibility of reducing vectorial competence using bacterial recombinant strains capable of releasing anti-parasite molecules.

## Background

Malaria represents one of the most devastating infectious diseases with an estimated annual incidence of around 200–500 million cases and 1–3 million deaths per year. Several factors contribute to the severity and worsening of this disease, among which the emergence of drug-resistant parasites and insecticide-resistant mosquitoes are of enormous impact [1]. Malaria parasites (*Plasmodium* spp.) accomplish part of their biological cycle inside the mosquito vector (*Anopheles* spp.) following a complex developmental program. A major bottleneck in the *Plasmodium* spp. cycle occurs in the midgut of mosquitoes, where gametocytes develop into ookinetes and then oocysts: from thousands of gametocytes ingested with a blood meal, only a few oocysts (usually less than 5) will develop [2-4], with some variations depending on the mosquito species as it is well documented in a comparative study in Tanzania focusing on *An gambaie* s.l. and *An. funestus* mosquitoes infected with *P. falciparum*[5]. This major bottleneck in the *Plasmodium* life cycle suggests that the ookinete/oocyst stage represents a target for the development of novel strategies for malaria control. Microorganisms associated with the midgut of mosquitoes might be applied to block the *Plasmodium* life cycle, and laboratory evidence has already been obtained supporting this possibility [6]. In addition, further stages along the *Plasmodium* development life-cycle might be targeted for malaria control, i.e. sporozoites after their emergence from oocysts, during their migration, and in the salivary glands. A strategy to exploit symbiotic microorganisms for the control of vector-borne diseases is paratransgenesis, i.e. the generation of engineered symbionts expressing anti-parasite molecules [7]. Different types of microorganisms have already been tested within laboratory conditions for their ability to block malaria parasites, through to the expression of a variety of effect or molecules [8,9]. The ideal candidate for the paratransgenesis control of malaria would be a microorganism that has a steady association with *Anopheles* mosquitoes, infecting not only the midgut but also other tissues where *Plasmodium* development occurs. This organism should also be amenable to cultivation, genetic manipulation and reintroduction into the mosquito populations. We have recently identified *Asaia* as a dominant symbiont of some malaria vectors [10,11], and have shown that this symbiont possesses most of the characteristics required for paratransgenesis applications against *Plasmodium* spp., however, major issues are still to be addressed in order to achieve a better understanding of the *Anopheles-Asaia* symbiosis. For example, *Plasmodium*-infected blood meals induce an immune response in mosquitoes that might limit not only the malaria parasite progression in the insect but also the symbiont population. Thus, the first question is whether *Plasmodium* interferes with *Asaia* presence/multiplication in mosquitoes. A related question is whether mosquito immune responses are effective on *Asaia*. Furthermore, in general, it is important to map the points of overlapping of the parasite (*Plasmodium*) and the symbiont (*Asaia*) in the mosquito body.

Using the malaria model *An. stephensi-P. berghei*, we show that the malaria parasite does not interfere with the growth of *Asaia* in mosquitoes and that the adaptation of *Asaia* to the insect immunity might explain its persistence even in mosquitoes heavily infected by the parasite. At the same time, *Asaia* is widely distributed in both the midgut and the salivary glands and sometimes it is even located in close association with the parasite at different stages of development in the mosquito. These characteristics of *Asaia* reinforce its potential for paratransgenic applications of malaria control.

## Methods

### Mosquitoes

*An. stephensi* samples were acquired from a colony reared since 1988 in the insectary of the School of Biosciences and Biotechnology (SBB) at the University of Camerino (Unicam), Italy. These mosquitoes were maintained in standard rearing conditions at a temperature of 29°C and 95±5% humidity, with a photoperiod of 12 h light/dark cycle. Adults were fed on mouse blood and a sterilized 5% sucrose solution.

### Malaria parasite

The malaria parasite used in the experimental infections was the recombinant strain PbGFP_CON_, a GFP-tagged recombinant strain derived from the murine pathogenic plasmodia *P. berghei* ANKA (kindly provided by Sinden R., Imperial College, London, UK). Strain PbGFP_CON_ constitutively expresses GFP at higher levels throughout the complete life cycle from a transgene that is integrated into the parasite genome [12]. The usage of PbGFP_CON_ allows visualization and investigation of live parasites at various stages of development in both mice and mosquitoes by fluorescent microscopy.

### Mice

Female BALB/c mice were maintained in the breeding facilities of the SBB at Unicam. Eight-week-old female mice were infected with *P. berghei* strain PbGFP_CON_ by acyclic passages through an intraperitoneal injection of blood from the tail vein of an infected mouse with around 10% parasitemia. About 5 μl of infected blood (~21×10^6^ infected erythrocytes) was diluted in 100 μl of 1× phosphate-buffered saline (PBS) at pH 7.2. Infected mice were monitored every couple of days for parasitemia by fluorescent microscopy as well as gametocytemia evaluation by through Giemsa stained blood smear.

### Ethical statement

All animal experiments were carried out according the Italian Directive 116 of 10/27/92 on the “use and protection of laboratory animals” and in adherence with the European regulation (86/609) of 11/24/86 (licence no. 125/94A, issued by the Italian Ministry of Health).

### Bacterial strains: GFP-*Asaia*, DsRed-*Asaia* and DsRed-*E. Coli*

Three recombinant bacterial strains expressing fluorescent proteins, GFP (Green Fluorescent protein) or DsRed (Discosoma Red), have been used: GFP-*Asaia*, DsRed-*Asaia* and DsRed-*Escherichia coli*.

The tagged strain GFP-*Asaia* was obtained from the native strain SF2.1 isolated from *A. stephensi* by cloning a gfp gene cassette in pHM2 plasmid [10] that confers resistance to kanamycin. The same native strain was also modified to obtain the DsRed-*Asaia*[13], in this case a mini-Tn5 gene cassette containing the dsRed gene was inserted into the bacterial chromosome by conjugation and transposition [14] and it is indefinitely retained without any antibiotic selective pressure.

The recombinant strain DsRed-*E. coli* DH5alfa pKan(DsRed) contains the dsRed gene expressed under the control of the ribosomal promoter rrnBP1 [15].

### Haemocyte cultures

‘In vitro’ experiments were performed using mosquito haemocyte cultures isolated from dissected *An. stephensi* adult females reared at SBB (UniCam) and macrophage-like *Drosophila melanogaster* embryonic haemocytes, SL2 cells.

### Mosquito infection with *P. berghei* and parasite counts

Quantitative analysis experiments were replicated on three different generations of mosquitoes maintained under the same rearing conditions. Each experimental set-up consisted of two cages, each containing a hundred female mosquitoes that seven days after emergence were fed with a blood meal from anaesthetised, infected or uninfected mice respectively. Infected blood meals were performed using mice showing a parasitemia around 10%. Unfed mosquitoes were removed from both cages, and then, the cages were housed in a chamber at 20°C and 95± 5% humidity, as this step is required for parasite development in laboratory conditions. Before and after the blood meal, mosquitoes were fed with a sterilized 5% sucrose solution. The control mosquitoes were tested just before the blood meal (five individuals per cage). At 24, 48 and 72 hours (h) after the blood meal ten mosquitoes were examined from each cage. The guts of the mosquitoes collected from both cages were dissected immediately after the sampling. Each single gut was placed in a sterile 1.5 ml “non –stick” low retention hydrophobic tube and carefully homogenized in 60 μl of sterile 1× PBS; low retention hydrophobic pipette tips were used to minimize malaria parasite loss during sample preparation. Half of the gut homogenate preparation was used for the parasite count. Ten μl of the gut homogenate was placed onto a slide and covered with micro glass (18×18 mm). The green early stages, ookinets and oocysts were counted using a fluorescent microscope Axio observer z1 (Zeiss) at 400× or 200× objectives, in triplicate.

### Quantitative PCR (qPCR) detection of native *Asaia* in *An. stephensi*

Quantitative analysis was carried out on the three experimental set-ups described above. Half of the gut homogenated was used for template preparation for qPCR analysis by Taq-Man probes. DNA was extracted from the guts immediately after the dissection by the Blood GenomicPrep Mini Spin Kit (GE Healthcare) and stored at +4°C in Dnase-free water. The DNA amount was determined using a Nanodrop (Thermo Scientific). Duplex PCR amplifications were carried out, each reaction contained 50 ng of template DNA in 2× PCR Brilliant Multiplex QPCR Master Mix (Agilent, Stratagene), passive reference dye Rox (1:500), two couples of primers (200 nM each) and two Taq-Man probes (200 nM each). Primers and probes used to amplify target sequences of *Asaia* 16S rRNA and *An. stephensi rps7* genes were obtained by Eurofins probe design [16] and a possible interference among sequences was checked by FastPcr program [17].

Bacterial primers and probe specificities were verified by ProbeMatch toll [18]. Sizes of *Asaia* 16S rRNA and *An. stephensi rps7* amplicons were 99 bp and 90 bp respectively. Amplification of the target sequence *An. stephensi rps7* allowed the normalization of an amount of *Asaia* that was estimated as a relative quantity, calculating the gene copy ratio (number of *Asaia* 16S rRNA/*An. stephensi rps7*). The probe sequences were labelled with the reporter dyes Hexachloro-Fluorescein-CE Phosphoramidite (HEX) or Cyanine −5 (Cy5) at the 5′ end (emission wavelengths 555 nm and 665 nm respectively), and the quencher dyes used were Black Hole Quencher −1 (BHQ-1) or Black Hole Quencher −2 (BHQ-2) respectively. Primers and Taq-Man probe sequences were:

*Asaia*-16S/for 5^′^TAGCGTTGCTCGGAATGACTGG3^′^,

*Asaia*-16S/rev 5^′^CGTATCAAATGCAGCCCCAAGG3^′^,

Rps7/For 5^′^AGCAGCAGCAGCACTTGATTTG3^′^,

Rps7/Rev 5^′^TAAACGGCTTTCTGCGTCACCC3^′^,

*Asaia*-16S/probe HEX-5^′^AAAGGGCGCGTAGGCGGTTTACACA-3^′^BHQ_1_,

Rps7/probe Cy_5_-5^′^CTACTGTGCGTCGTGGGAGATGAACGAA-3^′^BHQ_2_

The amount of amplified targets was measured by the development of standard curves obtained using templates of eight serial dilutions of plasmid preparations of both amplicons (from 2 to 2×10^-7^ ng). Standard curves had an average correlation coefficient of 0.999, a slope of −3.447, with an amplification efficiency of 95% in the case of *Asaia* 16S rRNA amplicon, and an average correlation coefficient of 0.996, a slope of −3.328, with an amplification efficiency of 99.7% in the case of *rps7*. Efficiency, sensitivity and specificity of the singleplex and duplex assays were comparable.

The thermal cycle was carried out under the following conditions: 10 minutes (min) at 95°C × one cycle; 30 seconds (sec) at 95°C, 1 min at 60°C and 30 sec at 72°C × 40 cycles.

### Statistical analysis

The statistical analysis of the amounts of *Asaia* was estimated by the qPCR assay, which was provided by the use of the Program R [19]. *Asaia* blooming after an infected or uninfected blood meal was analysed by ANOVA and post-hoc test ‘Bonferroni’ that uses a parametric analysis of variance (ANOVA/Bonferroni) based on Log_10_ values that follow the Gaussian trend required [20].

The correlation curves between *Asaia* amounts and the number of parasites counted have been assessed. The amount of parasites was considered as an independent variable (x) and the *Asaia* relative quantity as a dependent variable (y). Extreme values were estimated in the range of 3 standard deviations and the correlation was obtained using all data points [21]. Ten points were considered a significant population (n>9). Analysis of linear regression was performed by Student-t and Fisher tests.

### Mosquito colonization with recombinant *Asaia* strains

Regarding *Asaia/Plasmodium* co-localisation experiments, two cages have been set-up each one containing a hundred adult females fed with sugar solution, to which GFP- or DsRed-*Asaia* were added. For colonisation with GFP-*Asaia*, mosquitoes were fed with sugar solution containing 2×10^8^ recombinant bacterial cells/ml and 100 μg/ml of kanamycin during the first three days after emergence, while colonisation with DsRed-*Asaia* was achieved in the same way but without any antibiotic selection.

GFP-*Asaia* and DsRed-*Asaia* were grown for 24 h at 30°C in GLY medium, cells were harvested by centrifugation, washed three times in 0.9% (wt/vol) NaCl and adjusted to 10^8^ cells per ml^-1^ in 30 ml of H_2_O/5% (wt/vol) sucrose solution.

Seven days after emergence, the mosquitoes were provided with a blood meal from the same infected mouse in both cages, according to the same protocols previously described for quantitative analysis. Regarding the *Asaia/Plasmodium* follow up, five mosquitoes from each cage were collected for the dissection of midgut and salivary glands every two days until the 19th day after the blood meal. Midguts were fixed in 4% paraformaldehyde for 10 min at 4°C, whereas salivary glands were analysed on freshly prepared slides to avoid tissue deterioration. The slides were then mounted in glycerol-PBS for analysis by fluorescent microscopy using an Axio observer z1 (Zeiss).

Experiments were performed in duplicate for each recombinant strain.

### Semi-quantitative analysis by Reverse Transcription PCR (RT-PCR) of antimicrobial peptide (AMPs) expression ‘in vitro’

In relation to the induction of AMPs expression after bacterial challenge, *An. stephensi* and *D. melanogaster* immunocyte cultures were incubated with a 10^9^ cells/ml bacterial suspension (DsRed-tagged *Asaia* or *E. coli*) for 0, 4, 8 and 12 h. After treatment, cells were centrifuged at 800 g for 5 min at room temperature and the supernatant was discarded. Total RNA was extracted from cells using TRI-REAGENT TM (Sigma), following the method described by the supplier. Following extraction, RNA quality and concentration were assessed with a ND-1000 spectrophotometer (NanoDrop, Wilmington, DE, USA). RT-PCR was performed with the Access RT-PCR System (Promega), according to the supplier’s protocols.

Cytoplasmic actin was amplified as a loading control and PCR reactions (between 15 and 25 cycles) were carried out using the following parameters: annealing temperature 58°C, annealing time 40 sec, elongation time 45 sec. Even if a possible influence of immune challenge on actin expression was reported [22], we saw no differences in normalized versus actin results in either case. Concerning the defending and cecropin A, semi-quantitative PCR reactions (35 cycles), the following parameters were used: annealing temperature 61°C, annealing time 60 sec, elongation time 30 sec. In the case of drosomycin and the gambicin semi-quantitative PCR reactions (30 and 35 cycles respectively), the following parameters were used: annealing temperature 62°C, annealing time 50 sec, elongation time 30 sec. The amplification cycle numbers for the different reactions have been empirically defined for each product to examine the linear phase of PCR, during which template input was proportionally related to amplicon output levels.

The following primers have been used for the reaction:

Actin-F 5^′^AGCAGGAGATGGCCACC3^′^,

Actin-R 5^′^TCCACATCTGCTGGAAGG3^′^,

Defensin-F 5^′^GCTATCGCTTTTGCTCTGCT3^′^,

Defensin-R 5^′^CCACTTGGAGAGTAGGTCGC3^′^,

Cecropin-F 5^′^ACATCTTCGTTTTCGTCGCT3^′^,

Cecropin-R 5^′^CTTGTTGAGCGATTCCCAGC3^′^,

Drosomycin-F 5^′^CCGCAGTACCCACTCAATCT3^′^,

Drosomycin-R 5^′^ACTGCAAAGCCAAAACCATC3^′^,

Gambicin-F 5^′^AACCGGAAGGGGCGTTTCGT3^′^,

Gambicin-R 5^′^CGTCTGGCACTGATTAAACC3^′^

The expression of defensin, cecropin A, drosomycin and gambicin were evaluated after electrophoresis on 1% agarose gels. Gel documentation was collected using a “Gel Doc XR”, digitally evaluated with “Quantity One” (Bio-Rad Lab., Milan, Italy), and normalized to the correspondent signals for cytoplasmic actin. Seven replicates were carried out.

### Growth curves and phagocytosis test

Mosquito haemocytes were isolated from dissected *An. stephensi* adult females and maintained for 72 h in Schneider’s medium (Sigma, St. Louis, MO, USA), supplemented with heat-inactivated 10% FBS (Fetal Bovine Serum), 100 units/ml penicillin and 100 μg/ml streptomycin, before further analyses. Antibiotics were removed before phagocytosis and growth tests by centrifugation of cells and resuspension in fresh medium without any addition of antibiotics.

Macrophage-like *D. melanogaster* embryonic haemocytes, SL2 cells, were maintained at 25°C in Schneider’s medium, supplemented with heat-inactivated 10% FBS.

Recombinant strains of DsRed-tagged *E. coli* and *Asaia* were grown in nutrient broth for 48 h at 37°C and 30°C respectively.

For growth inhibition tests, cultures of *E. coli* (10^9^ cells/ml) and *Asaia* (10^9^ cells/ml) were heat-killed at 65°C for 45 min. The microorganisms were then centrifuged (4000 rpm for 10 min), washed in PBS and suspended in Schneider’s medium in order to obtain a final concentration of heat-killed bacteria of 4×10^8^/ml. Suspensions of both bacteria (4.5 ml) were added to mosquito cultivated haemocytes, at the final concentration of 1.8-2×10^6^/ml, and incubated for 24 h at 26°C. After incubation, the suspensions were centrifuged and the supernatants filtered. Medium obtained from mosquito cells exposed to bacteria, was tested undiluted and diluted (1:2) against *E. coli* and *Asaia*. The antibacterial activity of the conditioned medium, was registered by Biophotometer recorder (Biophotometer-Bonet-Mauri, Isa Biologie, France) for 30 h. Microorganism growth controls were performed in the Schneider’s medium.

In the phagocytic tests, haemocyte cultures from both mosquitoes and flies were incubated for 1 and 2 h in 5 ml of medium containing bacteria (*E. coli* or *Asaia*), and successively, the phagocytosis index was evaluated as the percentage of phagocytic haemocytes showing inside Ds-red labelled bacteria under an “Eclipse 90i” microscope (Nikon). The microscope was equipped with a super high-pressure mercury lamp and connected to a DS cooled camera head “DS-5Mc” regulated by “ACT-2U” software (Nikon). Ten phagocytic test replicated experiments were performed.

## Results and discussion

### Quantitative analysis of native *Asaia* and *P. berghei* within *An. stephensi*

The insect midgut is a major site of immune activity against pathogenic microorganisms [23]. In *Anopheles* mosquitoes, the ingestion of a blood meal carrying higher loads of *Plasmodium* is known to elicit a strong immune response, that acts both in the lumen (e.g. through the action of AMPs, nitric oxide and other effector molecules) and at the level of the gut wall [24]. The *Anopheles* midgut microbiota may be negatively affected by this response. Should the presence of a pathogen such as *Plasmodium* reduce the load of a given mosquito’s midgut symbionts, this would limit the utility of the symbiont itself for applications in paratransgensis. Furthermore, deciphering microbe-pathogen interactions offers new perspectives on disease transmission control [25]. Thus, we have investigated the kinetics of amounts of *Asaia* in mosquitoes, after supplying the insects with *Plasmodium*-infected or -uninfected blood meals. The experiments were conducted on three generations of mosquitoes using the murine malaria model *P. berghei*, with *An. stephensi* as a mosquito vector. The mosquitoes were examined using qPCR for native *Asaia* quantification. In particular, for each of the three experimental set-ups, 70 guts were sampled: 10 control guts (before blood meal) and 10 infected guts as well as 10 uninfected guts at 24, 48 and 72 h after the meal.

**Figure 1:**
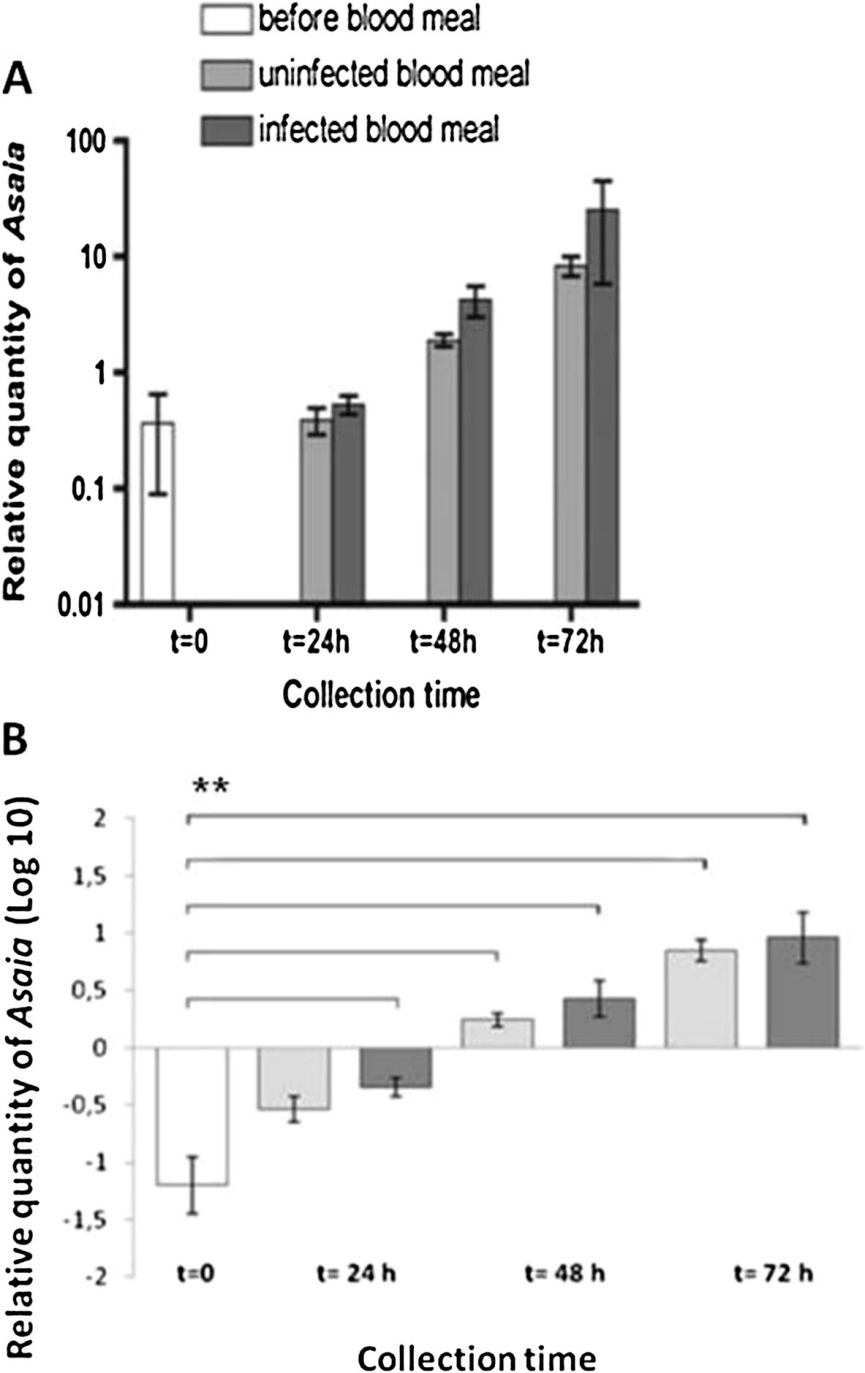
**Quantitative analysis by qPCR of the amount of native *Asaia *in *An. stephensi *midgut.** Mosquito collection time, after infected (dark grey bars) or uninfected (light grey bars) blood meal, has been fixed before (t=0) and 24, 48, 72 h after blood meals. The relative amount of *Asaia* was expressed as a ratio of bacterial 16S rRNA and mosquito *rps7* gene copies in logarithmic scale (panel **A**) and Log_10_ values (panel **B**); in both panels amounts were mean±SEM of ten individuals. Two asterisks in panel B denoted p<0.01 in the samples indicated by horizontal lines, compared by both ANOVA and Post-hoc test ‘Bonferroni’.

The bacterial load showed a growth after the blood meal, detectable at 24 h and increasing during the next two days, reaching about a tenfold quantity compared to the controls (p<0.01), in both *Plasmodium*-infected and -uninfected mosquitoes (Figure 1). Statistical analysis of three experimental set-ups revealed comparable trends in the replicates. Since, the amount of bacteria was sensibly changing among different generations similar to that shown in previous work [26], the results were not shown as an average of three replicates and only one representative data set was shown. Bacterial quantities were represented on a logarithmic scale to display highly increasing values (from t=0 to t=72 h): blooming of *Asaia* within the 24–72 h post blood meal was traced (Figure 1A). To focus on the differences between the control and the other samples, the means±SEM have also been reported by Log_10_ values (Figure 1B). There is, therefore, evidence that: i) *Asaia* blooms in the mosquito midgut after blood ingestion and ii) the bacterial blooming is not affected negatively by the presence of *Plasmodium in* the blood. We emphasize that the *Plasmodium* load in infected meals was very high, with about 10% infected erythrocytes. The growth of bacterial symbionts after the blood meal has already been reported in the case of other arthropods, including mosquitoes [27-29]. However, the evidence that *Plasmodium* presence at a high load does not interfere with the blooming of a bacterial symbiont (i.e. *Asaia*) is a novel result. Thus, the immune reaction triggered by *Plasmodium* does not interfere with the presence of *Asaia* in the insect. In summary, parasite infection in the mosquito does not seem to modify the kinetics of *Asaia* populations after the blood meal: the amount of bacteria in the midgut of infected mosquitoes can be considered at least comparable to that of uninfected specimens, and in considering all the cases, it is higher than in the control group.

**Figure 2:**
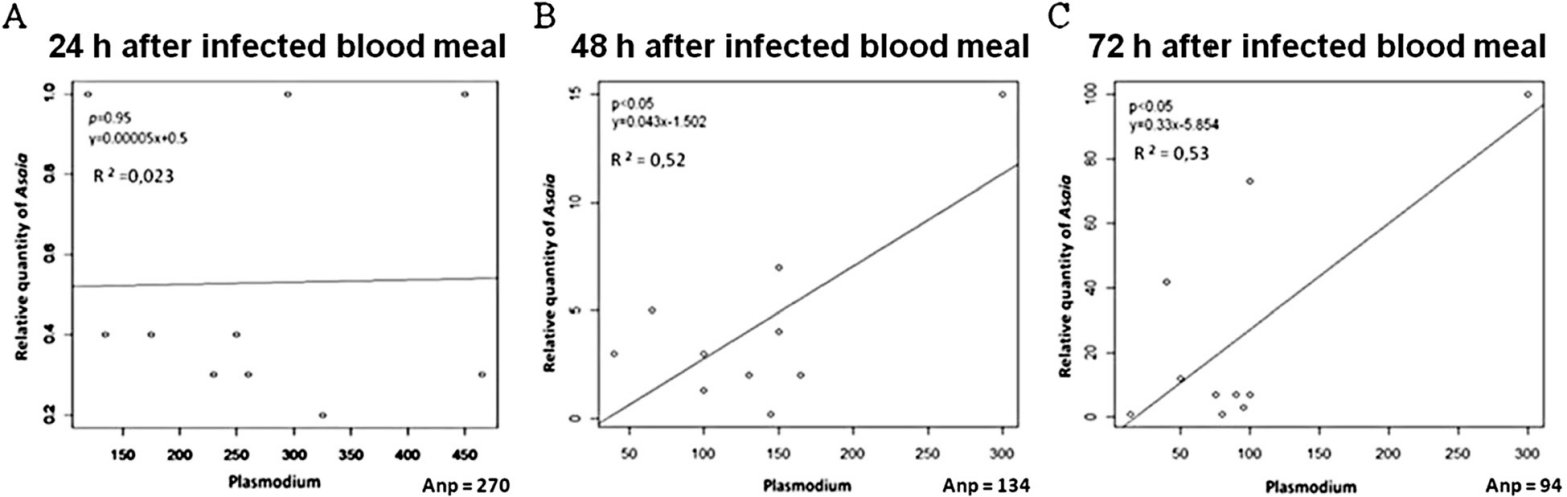
**Correlation curves between *Asaia *and *P. berghei *amounts in *An. stephensi *midguts.** Relative quantities of *Asaia* obtained from ten individuals presented on a logarithmic scale and the corresponding parasite numbers were reported at 24, 48 and 72 h after the infected blood meal (**A**, **B** and **C** respectively). The average numbers of parasites (Anp) and the curve slopes (Y) were shown. Analysis of linear regression revealed p values <0.05 obtaining a similar correlation curve (R^2^) at 48 h and 72 h.

In order to explain the quantitative analysis data in more detail, the number of parasites in the infected guts at 24, 48, and 72 hour post blood meal has been used to set up the correlation curves of *Asaia* and *Plasmodium* loads (Figure 2). Our data showed a drop in *Plasmodium* infection intensity during *Asaia* replication in the time period analysed: while the average parasite numbers dropped from 270 (24 h), to 134 (48 h) and eventually to 94 (72 h), there was an approximately eight-fold increase of *Asaia* at 72 h compared to 48 h (slopes ratio 0.33×/0.043×=7.67). Furthermore, the comparison between the amount of *Asaia* and the number of parasites in single guts revealed a positive correlation at 48 h and 72 h after *Plasmodium* challenge (p<0.05), obtaining similar correlation curves (R^2^=0.52 and R^2^=0.53).

Even though different phenomena could determine this correlation, we might perhaps suggest that the conditions in the mosquito midgut environment, that are more permissive for the development of *Plasmodium* oocyst, are also favourable for *Asaia* multiplication. Interestingly, these data showed that experimental infections leading even to one hundred oocysts, as is typical of the model used [30], are accompanied by conspicuous blooming of *Asaia*. This evidence, reasonably suggests a full compatibility of these two microorganisms for possible paratransgenic applications in nature, in fact, as it is known, the number of *P. berghei* oocysts in *An. stephensi* tends to be higher than as it is commonly observed in human *Plasmodium* infections, considering *An. gambiae* that rarely produce more than two-five oocysts [4,5].

### Interaction between *Asaia* and the mosquito immune system

**Figure 3:**
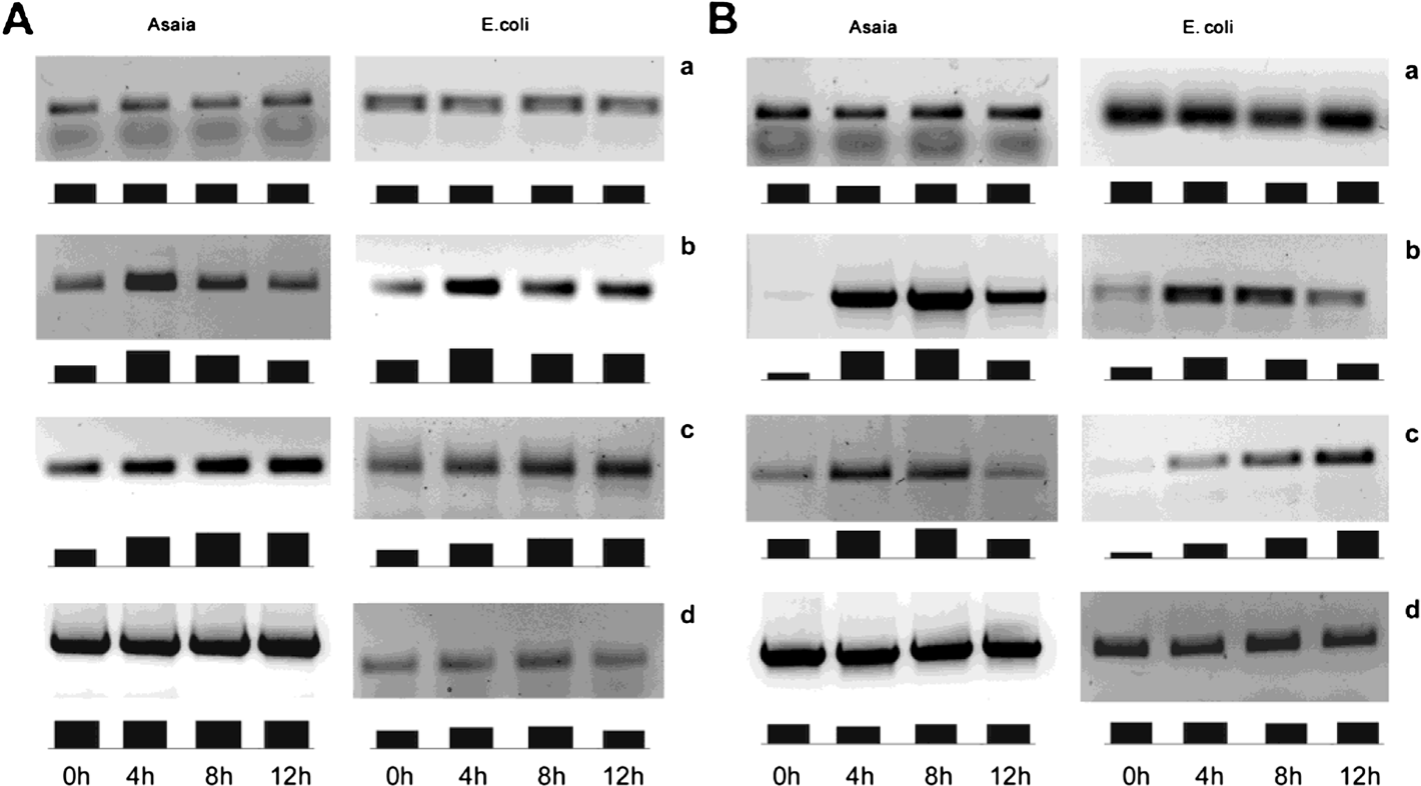
**Semi-quantitative analysis by RT-PCR of bacterial induction of AMPs in immunocyte cultures.** AMPs expression was evaluated in *An. stephensi* (panel **A**) and *D. melanogaster* (panel **B**). Defensin (a), cecropin (b), gambicin (c, panel **A**) and drosomycin (c, panel **B**) expression at 0h, 4 h, 8 h and 12 h after *Asaia* or *E. coli* bacterial challenge. Actin (d) has been used as a constitutive control gene. The expression of AMPs has been evaluated after electrophoresis in 1% agarose gel, documentation was collected using a “Gel Doc XR” and digitally evaluated with “Quantity One” as schematized below each panel. One representative set of data out of 7 replicates is shown.

**Figure 4:**
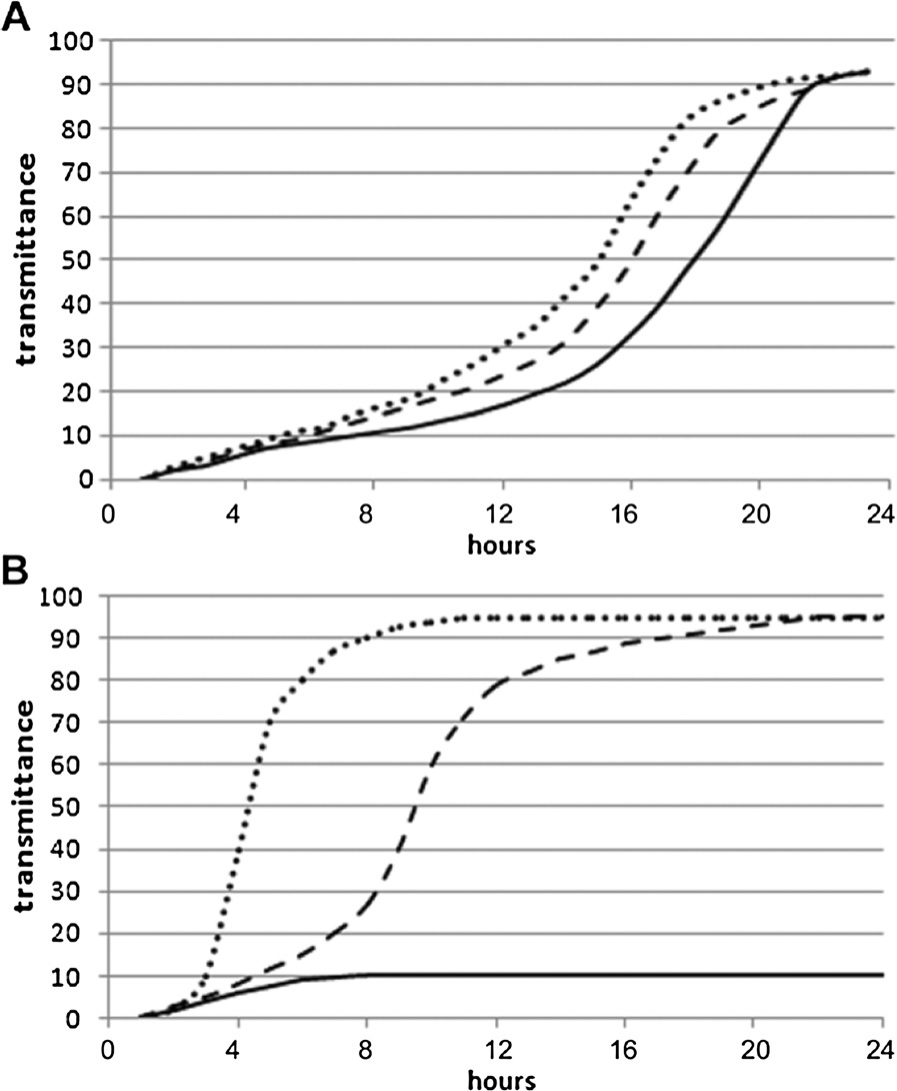
**Bacterial growth curves. ***Asaia* (**A**) and *E. coli* (**B**) were cultured in the presence of control Schneider’s medium (.....), or half-diluted (− − −) and undiluted mosquito haemocytes conditioned medium (^___^).

**Figure 5:**
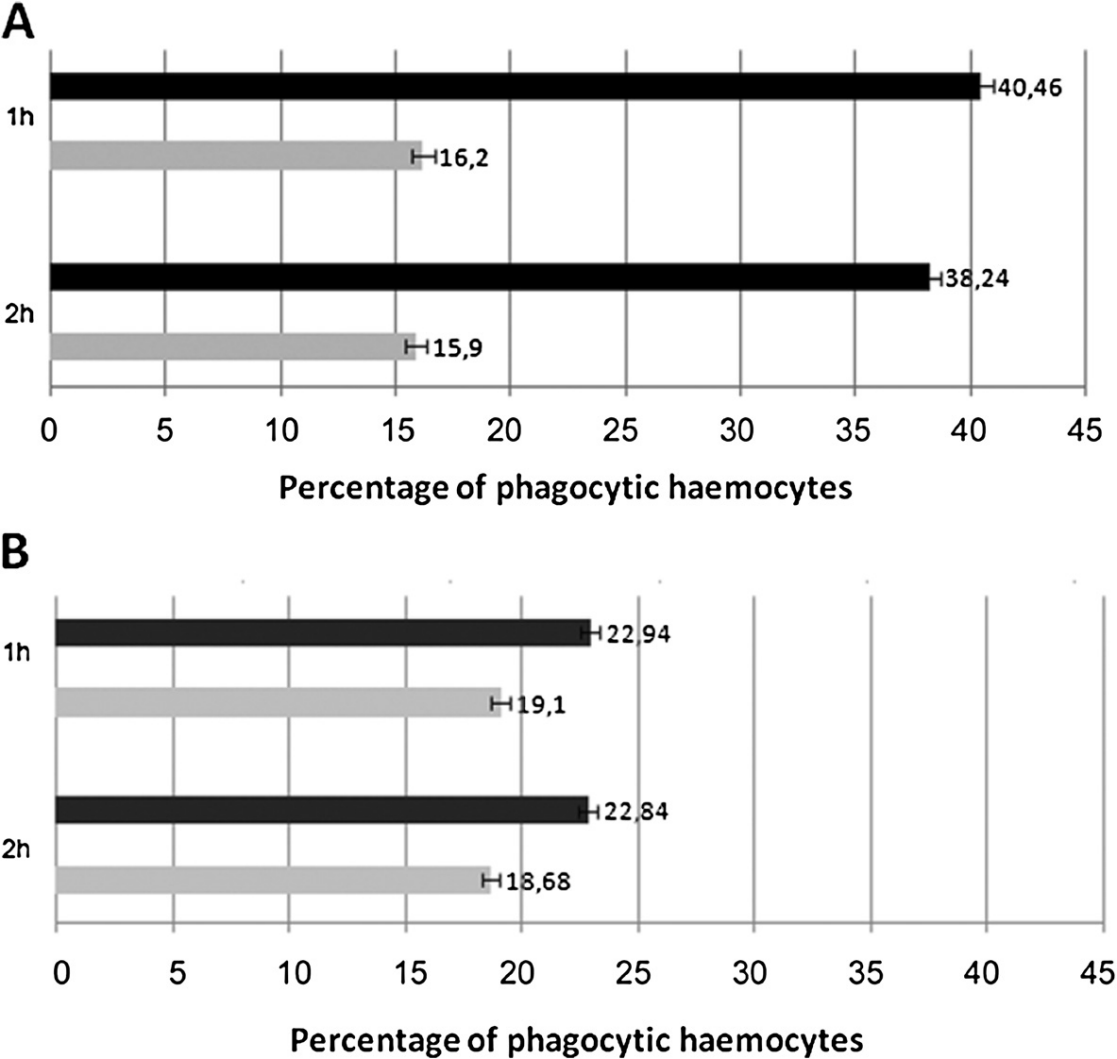
**Phagocytosis test analysis.** Phagocytic activity was evaluated in vitro using cultured *An. stephensi* (**A**) and *D. melanogaster* (**B**) haemocytes, and DsRed-labelled bacterial strains of *Asaia* and *E. coli*. The percentage of haemocytes showing fluorescent phagocytised bacteria, *E. coli* (black bars) or *Asaia* (grey bars), has been evaluated after 1 h and 2 h. Values are expressed as mean±SD of ten replicates.

The evidence that the ingestion of a blood meal carrying a high load of *Plasmodium* does not interfere with *Asaia* growth suggests that this bacterium is not affected by the immune reaction of the mosquito triggered by a malaria parasite challenge. Thus, we performed a series of experiments, with the final goal of determining whether *Asaia* is resistant to AMPs and phagocytosis. The expression of four AMPs (defensin, cecropin, gambicin and drosomycin) was investigated by semi-quantitative RT-PCR analysis on haemocytes from *An. stephensi* and *D. melanogaster*, after stimulation in vitro with *Asaia* or *E. coli* (Figure 3). Then, the growth curves of both bacterial strains were determined after exposure to conditioned culture medium of the mosquito haemocytes stimulated for the production of AMPs (Figure 4). Finally, we performed phagocytosis tests with haemocytes from *An. stephensi* and *D. melanogaster* in order to determine the efficacy of phagocytosis on *Asaia* using cells from the two insects in comparison with phagocytosis efficacy on *E. coli* (Figure 5).

The results of these integrated immunological studies can be summarized as follows: i) *Asaia* induces a comparable expression of cecropin, gambicin and drosomycin in both mosquito (Figure 3A) and fly (Figure 3B) cells, and the response is comparable to that determined by *E. coli*, and in particular, neither of these bacteria seemed to induce defensin gene expression, which has previously been described in the literature on Gram negative strains [31-33]; ii) *Asaia* growth is only slightly affected by a medium from a haemocyte culture stimulated for AMPs production, which suggests that these bacteria are, in some way, resistant to the mosquito immune effector molecules; iii) *Asaia* is less phagocytised than *E. coli* by both *An. stephensi* and *D. melanogaster* haemocytes, even though the difference is greater in mosquitoes in respect to flies.

Some differences between the capacity of *Asaia* and *E. coli* to induce expression of AMPs in *An. stephensi* haemocyte cultures could be expected since *Asaia* is stably associated with mosquitoes while *E. coli* has never been reported as a mosquito-associated bacterium. Nevertheless, our data support previous evidence showing that Anopheles bacteria induce mosquito antimicrobial immune responses [26]. Even though RT-PCR analysis did not indicate effective secretion of AMPs in the medium, the growth curves suggested the presence of antimicrobial molecules, revealing that *E. coli* multiplication is almost completely inhibited in the undiluted, conditioned medium, and is also partially inhibited in the diluted medium (Figure 4B). Interestingly, under the same growth conditions, *Asaia* is just slightly and only reduced in the undiluted, conditioned medium (Figure 4A). Therefore, the adaptation (or pre-adaptation) of *Asaia* to survival within the insect does not appear to be related to a reduced immunogenicity but with resistance to immune responses.

According to this observation, the results of phagocytosis tests also indicate that *Asaia* is, in some way, adapted to the life within the mosquito: the ratio of *E. coli/Asaia* phagocyting immunocytes was around 2.4 in *An. stephensi* (Figure 5A) and 1.2 in *D. melanogaster* (Figure 5B). Thus, mosquito haemocytes are half as effective on *Asaia* than on *E. coli*, while the difference using fly haemocytes is not significant (i.e. we might say that the fly haemocytes have almost the same efficacy on the two bacteria). Lastly, haemocytes either in flies or in mosquitoes have shown a similar phagocytic activity of *Asaia* within a range of 15-20% (Figure 5).

The results indicating that *Asaia* is well adapted to resistance to the immune reaction of two different insect species agrees with studies indicating the capacity of these bacteria to colonize phylogenetically distant insects. In addition, the evidence that *Asaia* is less phagocytised than *E. coli* by *An. stephensi* haemocytes (while fly haemocytes have almost the same efficacy on the two bacteria) supports the status of *Asaia* as a symbiont of anopheline mosquitoes.

### Co-localisation of fluorescent strains of *Asaia* and *P. berghei* within *An. stephensi* midguts and salivary glands

The quantitative analysis results indicated that *Plasmodium* does not interfere with native *Asaia* populations within the mosquito gut, even there the bacteria are thriving after the blood meal, usually at the stage of having completed the first part of parasite development (i.e. oocyst formation), that is, at least, comparable to that of uninfected specimens. Both quantitative and in vitro immunological experiments have shown that the host immune response, during the *Plasmodium* infection, while leading to a drop in parasite number, does not seem to be in conflict with *Asaia* replication. Then, it was also important to assess whether physiological changes induced by the parasite do not modify the spread of *Asaia* within the mosquito body. Here we demonstrated that, in infected mosquitoes, *Asaia* follows the same localization pattern (midgut and salivary glands) as in the absence of the parasite. With this aim, *An. stephensi* mosquitoes were ‘co-infected’ with fluorescent-tagged strains of *Asaia* and *P. berghei*, and the bacterial spread was monitored in a way as was performed in previous studies on uninfected specimens [10,11,13]. Following emergence, mosquitoes were fed on sugar solution containing one of the two recombinant bacterial strains: GFP-*Asaia* or DsRed-*Asaia* expressing green or red fluorescent proteins respectively. Then, the mosquitoes colonised with green- or red-labelled bacteria had a blood meal obtained from infected mice harbouring the strain PbGFP_CON_ that constitutively expresses the GFP in all the developmental stages of the parasite [12]. The use of two *Asaia* fluorescent strains, instead of one, allowed us to determine more easily the colonisation pattern within the tissues and to follow the dissemination of bacteria in the mosquitoes. The availability of two recombinant strains was very advantageous: (i) GFP-*Asaia* showed a brighter fluorescent signal compared to the red strain, thus it was more visible in the gut where the presence of very bright oocysts hid the signals of red bacteria; (ii) instead, red-tagged bacteria could be distinguished from sporozoites more easily than the green ones; (iii) the DsRed-*Asaia* strain allowed a better preservation of the salivary glands (in fact, this tissue became particularly damaged after a prolonged treatment of the mosquitoes with antibiotics).

**Figure 6:**
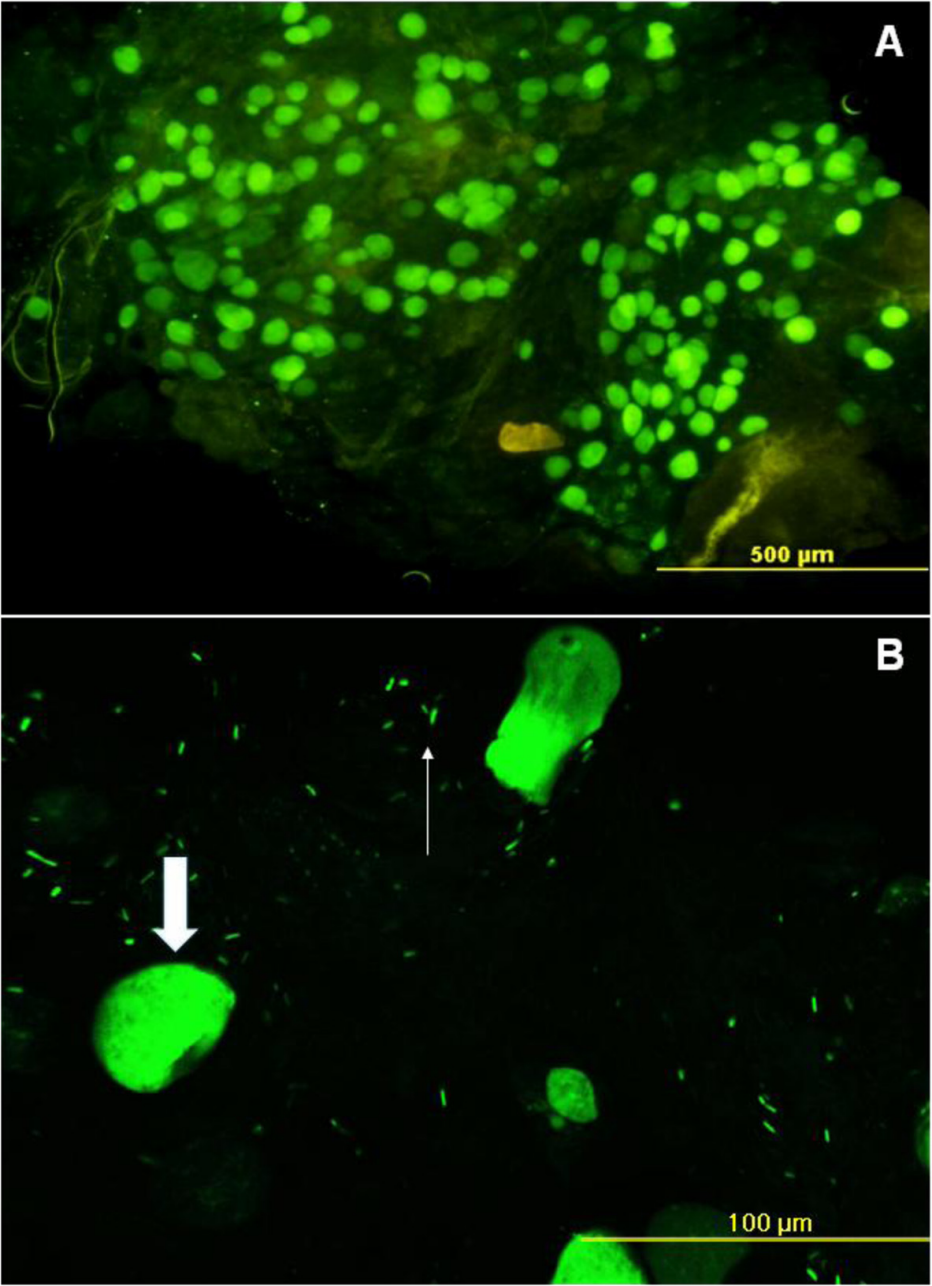
***Co-localisation *of GFP-*Asaia *and *P. berghei *PbGFP**_**CON **_**oocysts in the midgut of *An. stephensi*.** Microscopic fluorescence analysis was carried out 11 days after the infected blood meal and at the 15th day after bacterial administration. A massive presence of oocysts is evident in the midgut (**A**), parasite and bacterial co-localisation is appreciable in the magnified image showing the bacterium (thin arrow) as able to surround and overlap the mature oocysts (thick arrow) (**B**).

**Figure 7:**
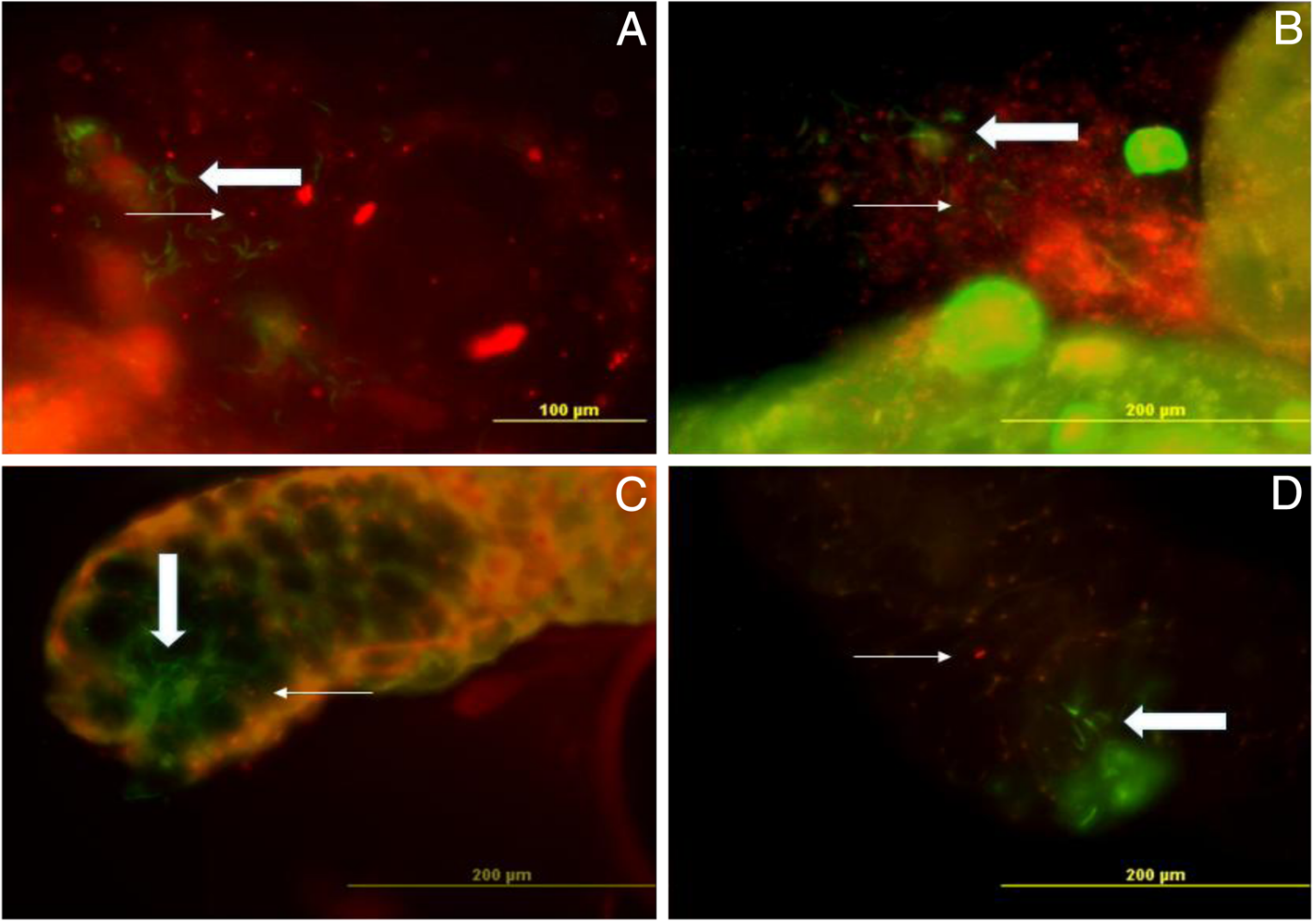
**Co-localization of DsRed-*Asaia *and *P berghei *PbGFP**_**CON **_**sporozoites in the proximity of mature oocysts and within the salivary glands of *An. stephensi*.** Microscopic fluorescence analysis was carried out on the 17th day after infection and at the 21st day after bacterial administration. The presence of red fluorescent *Asaia* (thin arrow) in the proximity of mature oocysts and GFP-tagged sporozoites (thick arrow) (**A** and **B**) as well as the co-localisation of the two microrganisms in the salivary gland lobes (**C** and **D**) can be detected.

Microscopic observations revealed fluorescent bacteria and parasites in the midgut showing that *Asaia* could also be located in close proximity of oocysts (Figure 6). Similarly, fluorescent bacteria surrounded sporozoites that leaked from mature oocysts and were found within the salivary gland lobes, as well (Figure 7). Thus, the presence of *Plasmodium* does not interfere with the displacement of *Asaia* that follows the same pattern already observed in uninfected mosquitoes. Furthermore, here we demonstrate that the two microorganisms clearly occupy the same organs where recombinant *Asaia* strains are widely distributed, and are even able to get in close contact with the parasite. It is a promising enterprise to propose the use of engineered bacteria to release anti-parasite molecules within the mosquitoes.

It is an interesting fact that fluorescent *Asaia* cells were observed to be still present in great numbers within the mosquito organs two-three weeks after bacterial administration (Figures 6 and 7). Microscopy observations, being consistent with quantitative data, revealed that *Plasmodium* as well as *Asaia* are able to complete their life cycle or are even capable of multiplication within the mosquitoes. The persistence of a fluorescent signal even after a considerable time after obtaining the marked strains demonstrated the presence of living and replicating bacteria. This observation is in agreement with the results of the immunological experiments (see above) indicating the adaptation of this microorganism to the host immune environment, even in the presence of strong stimulators of immunity, such as *Plasmodium*.

Moreover, the parasite infection does not seem to interfere with *Asaia* motility in the mosquito body. *Asaia* moves from the crop to other organs, but actually, we cannot yet establish the mechanisms of bacterial translocation. We had already demonstrated that recombinant *Asaia* strains supplemented by sugar meals are able to colonise the midgut and the salivary glands [10,11,13], however, this had not been demonstrated before in the case of malaria-infected mosquitoes. We have to mention that ookinetes migrate from the midgut lumen to the outer layer of the gut wall thanks to their active motility and molecular interaction with mosquito receptors [34]. Then from this location, sporozoites are released in the hemocoel and they migrate to the salivary glands. Here we showed that these events do not alter the localisation pattern of *Asaia*.

In conclusion, recombinant strains of *Asaia* are able: (i) to co-localise with the parasite in the midgut as well as the salivary glands; (ii) to surround *Plasmodium* in the different stages of development (oocyst and sporozoite); (iii) to preserve their vitality and mobility in infected mosquitoes. All of these characteristics are clearly suitable for paratransgenic approaches.

## Conclusions

In conclusion, if previous works denoted *Asaia* as a possible paratransgenic candidate for the control of malaria due to its stable association with anopheline mosquitoes, cultivability, transformability and capability of engineered *Asaia* to colonize mosquitoes by horizontal and vertical transmission routes, the results of this integrated study gathered and presented important information on the relationship of *Asaia* with both *Plasmodium* and *Anopheles*.

First of all, we revealed that physiological changes in the midgut following the blood meal do not negatively affect the residing *Asaia* population, on the contrary, the benefits of this condition were demonstrated by the bacterial blooming that occurred three days after feeding. Furthermore, the presence of *Plasmodium* in the blood meal does not interfere with the amounts of bacteria, allowing for the hypothesis that *Asaia* is able to evade the mosquito immune-response to the parasite infection, even under experimental conditions. This finding is a quite intriguing considering that naturally infected mosquitoes carry about one tenth of the number of oocysts than those reared. This means that, ideally, in wild infected mosquitoes, we would still have a relevant number of *Asaia* potentially able to exert a paratransgenic action against the parasite. However, when discussing possible implications concerning Malaria Symbiotic Control that arise from these observations, it must be considered that a potential difference between responses to *P. berghei* and *P. falciparum* in *An. stephensi* could occur just as it has been shown that *An. gambiae* immune responses to these two parasites are diverse [35,36].

Interestingly, while the parasite number is dropping, the bacterial amount greatly increases over time post-infection (8 times more at 72 h compared to 48 h). Nevertheless, we revealed a positive correlation between the number of *Asaia* and that of *Plasmodium* in infected mosquitoes, starting from the second day after infection. However, it is possible that this finding could be related to the high infection burden occurring under experimental conditions.

Secondly, we showed that *Asaia* can act as an immune-modulator microorganism in the mosquito activating the expression of some AMPs, even if it is not affected by these effects or massively phagocytised by haemocytes.

Even if our quantitative data did not find any correlation between the relative amounts of *Asaia* and *Plasmodium* within the mosquito midgut at 24 h, after the infected blood meal within the bounds of experimental infections, we cannot rule out that AMPs expression induced by *Asaia* may interfere with the parasite life cycle during invasion of epithelial tissues and translocation to the salivary glands [37,38]; for instance, an enhancement of ookinete lethality by gambicin has been observed [39]. *Asaia*, as well as other components of the mosquito midgut microbiota, may upregulate some immune genes, including several anti-*Plasmodium* factors. “*Asaia*-based” malaria control measure would not be negatively affected by immune reactions played out by the vector mosquito but it could rather reinforce that innate immune response of the mosquitoes.

Lastly, we showed that modified strains of *Asaia* are widely spread in the midgut and the salivary glands of infected mosquitoes, often being located in proximity of the malaria parasite. Therefore, exogenous recombinant bacterial strains are able to replicate in the mosquito body and to spread in the different organs for two-three weeks after the last administration, thus providing clear evidence that strains of *Asaia* releasing anti-*Plasmodium* effector molecules would be able to act against the parasite in the mosquito, reducing its vectorial competence.

All the results presented in this paper highlighted some important features of *Asaia* that would offer additional support to the feasibility of the *Asaia*-based malaria control strategies and presented a proof of the concept that attests this potential application in the field.

## Abbreviations

SBB: School of biosciences and biotechnology; PBS: Phosphate-buffered saline; GFP: Green fluorescent protein; DsRed: Discosoma red; h: Hours; qPCR: quantitative polymerase chain reaction; HEX: Hexachloro-fluorescein-CE phoshoramidite; CY5: Cynine-5; BHQ-1: Black hole quencher-1; BHQ-2: Black hole quencher-2; min: Minutes; sec: Seconds; RT-PCR: Reverse transcription polymerase chain reaction; AMPs: Antimicrobial peptides; FBS: Fetal bovine serum; Anp: Average number of parasites.

## Competing interests

The authors declare that they have no competing interests.

## Authors’ contributions

GF conceived the study and contributed to it with material collection, data analysis, interpretation and manuscript writing. AC, IR, CD, MM, PR, PS, EC, SE, MV, MM, LS provided qPCR and RT-PCR analysis, confocal analysis and immunological studies. MA’s contribution was statistical analysis. CB and DD contributed to data interpretation and manuscript writing. All authors read and approved the final version of the manuscript.
